# Adolescent to adult health care transition for persons with intellectual and developmental disability: current barriers, next steps

**DOI:** 10.3389/fped.2025.1486325

**Published:** 2025-08-15

**Authors:** Shwetha Ramachandran, Solveig Stensland, Julie H. Corder, Carrie Cuomo, Thao-Vi Dao, Marvin R. Natowicz

**Affiliations:** ^1^ Cleveland Clinic Lerner College of Medicine, Case Western Reserve University, Cleveland, OH, United States; ^2^ Children’s Institute, Cleveland Clinic, Cleveland, OH, United States; ^3^ Department of Internal Medicine and Geriatrics, Cleveland Clinic, Cleveland, OH, United States; ^4^ Pathology & Laboratory Medicine, Genomic Medicine, and Neurological Institutes, Cleveland Clinic, Cleveland, OH, United States

**Keywords:** intellectual disability, developmental disability, intellectual and developmental disabilities, health care transition, adolescent to adult, pediatric to adult

## Abstract

The transition of health care from adolescence to adulthood is a challenging time, especially for persons with intellectual and developmental disability (IDD), where navigating health care transitions can be particularly difficult. Persons with IDD are an especially vulnerable population and they and their caregivers encounter barriers in obtaining high quality health care transition. These barriers result in the suboptimal utilization of health care transition services and consequent poorer health outcomes. Herein, we discuss barriers to obtaining high quality pediatric to adult transitional health care for persons with IDD. We then discuss next steps, some of them well recognized and others underappreciated, for addressing these barriers and thereby achieving an important public health need: the attainment of high quality pediatric to adult health care transition for persons with IDD.

## Introduction

The health care transition from pediatric to adult health care is an important aspect of the provision of good health care. The seminal reports, “Supporting the Health Care Transition from Adolescence to Adulthood in the Medical Home”, provide a framework for preparing adolescents for transition, beginning between ages 12–14 and concluding when patients are between ages 18–26 (1, 2). This framework emphasizes a planned process for youth and young adult empowerment, self-determination, and self-management and the achievement of successful integration into the adult health care system.

The process of pediatric to adult health care transition is important for all adolescents and young adults and can be a challenging process for many. Studies on transition outcomes, while of variable quality, indicate that structured transition interventions often result in positive transition-related outcomes; conversely, an absent or unstructured health care transition experience is associated with care gaps, poorer health and increased rates of hospitalization (3–7). Yet, in the U.S., only about 20% of youth receive necessary health care transition preparation (8).

Persons with intellectual and developmental disabilities (IDD), a population group that is variably defined (9) but whose “membership”, however constructed, is characterized by multiple vulnerabilities across the lifespan, including the receipt of good quality health care (10–12). The successful transition to adult health care is especially needed but is often particularly challenging for youth with IDD as they have unique health differences and health care needs relative to their peers in the general population. Intellectual disability, if present, may affect one's ability to fully participate in health-related decision making and navigate the transition process and the adult health care system. Additionally, youth with IDD are more likely to have one or more serious medical, neurological and/or mental health co-occurring conditions (13–17). These factors render young adults with IDD particularly vulnerable to care gaps. Absent or poorly integrated health care transition in some instances may be the cause and in many instances is likely contributory to the development of multimorbidity for adults with IDDs (17–22).

For many reasons, described below, most adolescents and young adults with IDD do not receive formal or adequate transition planning (23–25). In this Perspective essay, we provide an overview of the many barriers to obtaining high quality pediatric to adult health care transition for persons with IDD and then discuss approaches for addressing these barriers.

## Challenges in pediatric to adult health care transition services for persons with IDD: key parties and conceptual framework

In discussing the challenges and frank barriers involved in obtaining high quality pediatric to adult health care transition services for persons with IDD, it is useful to begin by considering the diversity of the people accorded this designation. The spectrum of adaptive and intellectual functioning and associated medical and non-medical needs of youth with IDD is extensive (13–17). The designation of IDD encompasses persons with a vast range of phenotypes in the areas of cognition and communication, behavior, sensory deficits and needs, mobility and self-care skills, as well as a large spectrum of medical therapies and requirements for technology supports. Similarly, it includes persons with a vast range of hopes and dreams for their lives. Some of the conditions within the IDD designation are inherited, others acquired; some are present prenatally or around the time of birth and others present later in childhood. Some are associated with static neurodevelopmental courses, whereas others are associated with developmental or neurological regression. The diversity of phenotypes contained within the designation IDD creates significant challenges when developing care strategies for this population. For example, the spectrum of cognitive and communicative abilities, ranging from non-verbal status with profound intellectual disability to persons with mild intellectual impairment and highly fluent communication skills, requires different adaptations to meet the health care transition needs of patients and their families. There similarly is a broad spectrum of behavioral phenotypes as well as physical and medical differences and associated needs. Consequently, any program for the transition of care for persons with IDD from adolescence to adulthood should account for the varied cognitive, adaptive, and medical phenotypes of those with IDD or of the subpopulation of those with IDD served by their program, as well as their non-medical interests and needs, thereby facilitating a person-centered approach to their care.

There are multiple parties central to the health care transition process for persons with IDD. Some of the principals include the family members or guardians of persons with IDD (henceforth, caregivers), primary and specialty clinical care providers, clinical and administrative support staff, allied health professional specialists, care coordinators, social workers, legal professionals, hospital administrators and still others. The perspectives of all of the parties noted above are vital for the design and operation of effective health care transition programs. The incorporation of the diverse perspectives of these different stakeholders not only facilitates a person-centered approach to care for youth and young adults with IDD but also supports important family- and systems-related needs.

There are varied ways to analyze the challenges involved in achieving high quality health care regarding pediatric to adult transition for persons with IDD. The approach used here is to note the challenges when viewed from the perspectives of three groups of principal stakeholders: the adolescents/young adults with IDD, their caregivers, and their clinical care providers. We find considerable overlap in the barriers that they describe. We then reframe these challenges into two broad categories: system of care level barriers and societal level barriers. Finally, we discuss next steps, some of them well recognized and others underappreciated, for addressing these barriers.

## Barriers to quality pediatric to adult transition health care: the perspectives of participating youth and young adults with IDD

This level of analysis refers to the personal challenges that individuals with IDD encounter as they transition from pediatric to adult health care services. A large variety of challenges have been voiced. Reported challenges, stated by persons living in different regions and who receive care through varied clinical systems and who have diverse physical and neurodevelopmental phenotypes, include complaints of incomplete understanding of the transition process, low expectations of themselves, perceptions of low expectations by others, feelings of abandonment as they transition from pediatric providers, and a desire for and sometimes conflict in seeking decision making independence from caregivers. They also reported inadequate involvement in the health care planning process, a lack of information about the transition process and related resources, difficulties forming relationships with new providers, limitations in self-advocacy skills needed to navigate adult medical culture, fatigue from seeing doctors or from seeing too many doctors, inadequate supports in the transition process and for self-management, a lack of coordination of the transition process and in adult health care services, a lack of expertise of some of their health care providers, challenges related to transportation, and challenges with insurance/financial matters (26–35). Racial discrimination has also been voiced as an important impediment in transition health care (36).

It is important to recognize that the barriers that individuals with IDD face while navigating the health care transition process are always superimposed on their daily life experiences. For many this includes daily challenges related to physical or mental health differences, as well as possible challenges related to family dynamics, poverty, discrimination or still other factors.

## Barriers to quality pediatric to adult transition health care: caregivers' perspectives

This level of analysis refers to the problems that caregivers face in assisting their children's transfer of care to adult health care services. While many of the reported challenges are also voiced by patients, some are specific to caregivers. The challenges noted below are reported by parents caring for children with diverse forms of IDD from diverse health care systems and countries.

Caregivers voiced numerous difficulties pertaining to their children's health care transition process, including experiencing significant emotional and physical health stresses. Many studies noted that they reported a sense of abandonment by the health care system and feelings of both isolation and of vulnerability of their children (27, 37–39). Some mentioned the burden of additional competing obligations to others in the family or impacts on their own health, work and still other aspects of their lives (37, 38, 40, 41).

The stresses they reported related to the shortcomings of the transition process and of the adult health care system for their children. There were many reports of inadequate provision of information regarding the transition process (27, 39, 42, 43) or that the transition planning took place at too late a time (44). Inadequate information about financial considerations in the transfer of their child's health care to the adult medical system or the expense of needed care for the young adult were also noted as barriers to accessing services (43).

Issues regarding medical decision making for young adults with IDD can be complex and some caregivers also reported not receiving adequate information regarding these issues (44). Related to this, some caregivers reported concerns about relinquishing control over the medical care of their young adult children, worrying that their children may not be able to adequately manage their care. The complexities of transitioning medical decision making to the young adults with IDD was sometimes a source of tension between different dyads of stakeholders (43). For some, the achievement of independent medical decision making for young adults with IDD was not uniformly held to be as high a priority as other objectives of the transition process; this presumably largely related to limited cognitive function of those having severe or profound intellectual disability (45). Some caregivers voiced feelings of frustration associated with a perceived lack of respect for them and their expertise in caring for their children (38, 40, 42, 44).

Most studies indicated parental frustration of the inadequacies and inefficiencies of the services needed for the care of their children. One frequently voiced critique was insufficient coordination of the transition process for the patient and fragmentation of his/her clinical care, sometimes accompanied by poor coordination among adult medical services (37–39, 41, 43–45). This was described as a health care culture shift for their children, from their previous more holistic care centered in a pediatric medical home to a more episodic and reactive mode of adult care. Many caregivers reported needing to serve as a transition coordinator for their children, some even having to battle for services during the health care transition process that were essential for the health of their children (38, 41). Caregivers cited inadequate resources within these systems to meet the complex needs of their children including insufficient equipment and facilities and difficulties in accessing primary care and medical specialists knowledgeable or willing to care for their children with IDD (27, 37, 38, 41); these concerns were amplified in the case of some rare disorders and for families in rural areas that had shortages of providers and programs (43, 44). Caregivers for those with rare disorders associated with IDD noted that the state of incomplete knowledge about the natural history and treatment of many of these conditions adds additional complexity in the care of their children (44).

The critiques of the pediatric to adult health care for their children with IDD extended beyond the medical system. Concerns were voiced about the functioning of the medical, education and community systems as separate silos having minimal collaboration with each other, despite needing to work in an integrated manner to adequately provide for the needs of the young adults with IDD (39, 40, 44). Young adults with IDD were sometimes “left floundering” in adulthood for considerable periods of time, waiting for receipt of adult services or other community supports (27, 37–40, 42–44).

Analogous to the health care transition experiences of their loved ones with IDD, the challenges experienced by caregivers can be exacerbated (or mitigated) by other family, community, and societal factors. Some caregivers specifically noted the value of informal support networks or formal parent support or disease-specific support groups in providing emotional support and/or useful information about the transition experience (37, 40).

## Barriers to quality pediatric to adult transition health care: clinical care providers' perspectives

Clinical care providers in the pediatric to adult health care transition process are a diverse group of clinicians and include primary care and various clinical specialty providers. Like the other major stakeholders in this process, providers report multiple challenges in accomplishing effective transition services for persons with IDD. Some providers noted insufficient knowledge of both the transition process and of the unique needs of persons with IDD that are required to adequately engage their clients in the transition process. Salient examples include insufficient knowledge or comfort with clinical management or guardianship-related matters (32, 46). These limitations can be particularly pronounced in the clinical management of youth or young adults with IDD with medical and/or mental health complexity.

Clinical providers also reported that there are insufficient numbers of both primary and specialty health care providers with training or expertise in treating patients with IDD and associated conditions (34, 46–48). This problem of insufficient availability of appropriate clinicians to serve IDD youth can be exacerbated in rural and other medically underserved areas. An additional state or country-specific concern is the difficulty in accessing appropriate generalists or subspecialists because of various health insurance barriers. Many persons with IDD in the United States, for example, are served by public insurance and this, in turn, limits access to many clinical care providers, especially for adults with IDD.

Persons with IDD may have challenges in understanding complex information and in expressing themselves and may also have complex medical histories. These issues necessitate increased time for medical encounters. In usual medical practice, however, clinicians report that they typically are not provided additional time during clinic sessions for this patient population (39, 46, 48). Moreover, while optimal clinical care for medically complex patients requires consultation and coordination with multiple medical services, some medical reimbursement systems, such as in the United States, have hurdles in enabling financial coverage for meetings involving multiple clinicians (39, 46, 48). This issue extends to other needed non-medical consultations that are important in enabling effective transition programs, such as social work and legal consultations for the affected individuals and their family members. The substantial financial debt incurred by some clinicians during their training can also impact their willingness to be devoted to caring for patients with IDD whose care may be less remunerative and associated with other perceived challenges (48).

Providers also noted various educational and administrative barriers that are imposed on their clinical practices. For some this included insufficient or suboptimal educational materials for themselves or their patients, difficulty with electronic medical record systems in including relevant information, as well as difficulties in communicating with clinical care providers working in different medical systems (32, 39, 46, 49, 50). Some noted a need for standardized surveillance and management guidelines in their fields (47, 48). An inadequacy of relevant training for medical students and training programs for residents, fellows, and established practitioners in many clinical fields was also reported (47–49). Providers also reported insufficient administrative and financial support for pediatric to adult transitional health care efforts by the hospitals or clinics that they practice in or by their governments (47, 50).

Some clinical care providers cited family- and patient-related barriers to achieving effective transition. Examples included difficulties or resistance to transition by patients or caregivers because of strong bonds with the pediatric service or fear of leaving the pediatric system; economic issues of families and patient crises were other factors that could be barriers to effective clinical transition (46, 48, 50).

## System of care and societal level challenges to obtaining quality pediatric to adult transition health care

The barriers to obtaining quality pediatric to adult health care transition experienced by patients, their caregivers and their clinical care providers can, with only a few exceptions, be largely categorized into two broad areas: barriers at the system of care level and societal level barriers. System of care level barriers refer to barriers that are inherent within various systems of care that impede the transfer of pediatric to adult health care for persons with IDD. Pertinent systems of care include health care systems and aspects of educational and legal systems. These systems of care exist in the context of local, regional, and national governmental resources and regulations that are specific to the location of the affected individual and their family. Depending on the patient's location, therefore, there may be greater or lesser access to associated important resources, thereby impacting both medical and non-medical dimensions of life of the individual with IDD.

Societal level barriers refer to barriers that are a “higher” level of barrier. They are pervasive throughout systems of care and are embedded within the social, economic and political structures of a society. Important societal level barriers include poverty, income inequality, various forms of systemic discrimination and bias and geography-related barriers.

Numerous and varied system of care level barriers were voiced by persons with IDD, caregivers, and clinical care providers. These are noted in the previous sections of this essay and listed in Supplementary Table S1, along with their known or proposed causes and proposed interventions for their amelioration. In general, the system of care barriers include limited availability of structured pediatric to adult health care transition programs, problems with access to care for persons with IDD due to health care provider shortages or inadequate expertise of clinical providers, inadequate time for the clinical encounters, inadequate information and other resources to support the transfer of care for persons with IDD and their families, inadequate coordination of information and of clinical care within and between systems of care, inequitable access to office care related to scheduling or the physical design of clinics or transportation to clinics, financial challenges relating to the coverage of clinical care and related services that impact patients and caregivers, and financial challenges that relate to professional remuneration and that impact clinical providers and their administrators and clinics/hospital system.

There also are diverse, important societal barriers to pediatric to adult health care transition, including but not restricted to those with IDD. These are especially apparent through formal epidemiologic analyses and are also listed in Supplementary Table S1. Socioeconomic differences, the language used in the household, sex, and race/ethnicity impact health care transition disparities for youth (8). Low-income adolescents in the U.S. with IDD have been reported to receive less health care transition services compared to higher income peers (24, 25). Minorities, particularly Black and Hispanic patients, are also less likely to receive health care transition services, compared to white peers. Black individuals with IDD cite other systemic inequities, including inadequate diagnostic evaluations and biases within schools and other public programs, as further barriers to care (25, 36, 51). Provider bias and discrimination based on a diagnosis (or perception of the diagnosis) such as a mental health diagnosis in persons with IDD has been noted as well (34). Related studies indicate that the receipt of health care transition services varies substantially for youth with different chronic conditions such as intellectual disability, autism or cerebral palsy relative to persons with other special health care needs (23, 24). Disparities in care that relate to geography, such as particular difficulties regarding access to needed medical care for persons living in rural areas, have been reported. Patients having rare conditions who need access to specialty care that has limited availability and which is often only present in medically resource-rich areas, patients living in remote areas, and patients lacking access to appropriate transportation are particularly vulnerable to geography-related health care disparities (43, 44).

An alternative, additional perspective by which to consider many of the barriers experienced in the health care transition process is that they are consequences of ableism (52, 53). This conceptualization views many of these challenges consequent to past and present policies, institutions and norms that devalue and disadvantage persons who are disabled. It can therefore account, in part, for barriers ranging from inadequate resources for all stakeholders in the transition process, inadequate training for health professionals around working with disabled persons, transportation-related challenges for patients and caregivers, difficulties with communication access, limitations regarding the accessibility of scheduling platforms, limitations of the physical accessibility of facilities and more (52–54).

## Moving forward

These system level and societal level barriers to pediatric to adult health care transition, often leave youth and young adults with IDD unserved or underserved and they and their caregivers unduly burdened with orchestrating a complex transition process. In addition to detailed resources regarding the principles and practical operation of health care transition programs (2, 55), there are excellent, user-friendly resources for health care transition for youth with disabilities, including IDD, that are separately targeted for youth/young adults, parents/caregivers and clinicians/direct service providers (56). Moreover, multiple parties have issued thoughtful recommendations regarding how to remedy the barriers in health care transition experienced by persons with IDD or complex health conditions (2, 5, 44, 57–64). Previously reported or proposed initiatives to remedy these barriers, as well as some of our own, are noted in Supplementary Table S1. Rather than repeat those recommendations that are excellent and generally agreed upon, we briefly discuss several perspectives on solving transition-related barriers that, in our view, are inadequately emphasized in the literature. We then briefly note some underemphasized opportunities to improve aspects of the health care transition process for those with IDD.

### Moving forward: some important but underemphasized perspectives

First, on creating or implementing a change in a health care transition program, the insights and needs of all key stakeholders of the transition program ecosystem should be recognized and met for a highly functional, sustainable program to be achieved. To accomplish this, it is essential that all key stakeholders in health care transition programs be meaningfully involved in decision-making processes. This should include persons with IDD to the degree that they are able to participate. The process of involving persons with IDD in the medical decision making process can be complex, depending on the presence and extent of intellectual disability and/or communication impairment and the abilities of caregivers and clinical providers to facilitate the maximum involvement of each patient. The transition program needs to be involved in this process (2, 65).

With respect to the caregivers, their insights about the transition process and their health and wellbeing are also essential. They have special insights about the medical and non-medical needs of their loved ones and participate in their care and transportation; consequently, their input is essential. And their health and other aspects of their lives are intertwined with their children's lives and health care transition; some quality assessments of the transition process include caregiver wellbeing among their parameters (7).

Clinical care providers on both the pediatric and adult sides of the transition process interface with the patients and their families, social workers and hospital administrators and the insights of these providers are therefore invaluable. Clinicians need adequate time to provide good quality clinical care, good communication systems with patients, caregivers and colleagues, adequate clinical space, appropriate clinical equipment and administrative supports.

Hospital administrators make key decisions regarding personnel, allocation of resources and space issues affecting transition programs. Consequently, hospital administrators need to be informed of the goals, needs and progress of the transition programs and, in turn, need to provide feedback from their perspective.

Providing a forum for all stakeholders to meet and collaborate is essential to the success of a transition program. Different agendas and different types and frequencies of meetings are needed to achieve the needs of all stakeholders. Some committees/agendas speak to the needs of a specific patient/family, others relate to the “global” design and function of the transition program. The particulars of the committees and the frequencies of their meetings will necessarily be context dependent; one size will not fit all transition programs. Yet, regardless of whether the agenda pertains to a specific individual or to the general functioning of the program, there must be meaningful representation of all major stakeholders in decision making processes. The perspective noted here is different than the frequently cited need for “patient-centered” care. We endorse patient-centered care but all major stakeholder needs should be met for a transition program “ecosystem” to be effective and stable over time.

Second, there is still another important layer of complexity in the attempt to have good representation and communication of the major stakeholders in the health care transition process. The foremost priority of a health care transition program is to ensure effective pediatric to adult health care transition. However, all stakeholders recognize that a healthy, comprehensive transition from the pediatric to adult years is not limited to the patient's medical needs but relates as well to the education, vocational or day program, and social/recreational needs of the patient. This, in turn, necessitates at least minimal integration of the special education system, community organizations and local government with the health care transition process. The specifics of such integration will depend on the needs of each patient and the resources of each of the parties and the region/country where this takes place. Nevertheless, it is extremely important in the life of the patient.

Third, a review of the causes of the diverse barriers to health care transition listed in Supplementary Table S1 indicates that a disproportionate fraction of the barriers is, in our view, attributable to insufficient financial resources and other supports. The basis for this is multifactorial and specific to each region/country but often results from the low reimbursement of many clinical and administrative services of transition programs. Regardless of whether every phase of a transition program is well designed, it must have adequate financial and other resources and those resources need to be stably available for that program to be effective in the long term. The development of adequate and stable financial backing for transition programs is complex and requires commitment by hospital administrators, public health policy makers and legislators. It is a fundamentally important issue that is discussed in only a minority of the documents on interventions to improve transition programs—with some very important exceptions (e.g., 2)—but impacts nearly all such programs, albeit less so in medically well-resourced areas served by universal health care systems. Insightful strategies to improve the financial wellbeing of U.S. health care transition programs have been proposed (2).

A fourth high level perspective on issues pertaining to barriers in health care transition is the analysis of many such barriers as being consequent to ableism, as mentioned above. Viewing transition-related barriers in this light also suggests multiple strategies for intervention; some of these involve regulatory and legislative remedies that would likely be location specific (Supplementary Table S1) (52, 54).

Still other actionable transition-related barriers are relatable to ableism. The literature on remedying barriers to health care transition for those with IDD speaks to inadequate numbers of clinicians who are knowledgeable in caring for those with IDD and the need for improved medical education in this regard. While this is true, this gap in health professional education is part of the larger problem of the inadequacy of health professional education regarding disability-related matters more generally (66, 67). This is partly attributable to ableist perspectives in health professional education. Learning in these areas will expand the pool of clinicians competent in caring for those with IDD (and other conditions) and engender deeper understandings of the lives of those with disability for all clinicians.

The literature on remedying barriers to health care transition provides only minimal discussion of issues of transportation to and the physical space of clinical programs. These are issues that relate to the needs of both the patients and families as well as the needs of the clinical staff and are consequent to, at least in part, ableism. In terms of the former, there should be attention to accessibility of the clinic by public transportation and increased use of telehealth, when appropriate. Accessibility of the clinic rooms and other spaces for persons with mobility or other relevant physical limitations is essential. There should also be modifications to accommodate those with special sensory limitations or sensitivities. The clinic space should be equipped with accessible scales, accessible examining tables and other equipment needed to allow for safe and comprehensive examinations of patients; there are recent federal regulations in the U.S. regarding this matter (68).

### Moving forward: some underemphasized or relatively new opportunities to improve transition-related barriers

It is important that health care transition programs, like any health care initiative, have quality assurance/quality improvement processes and therein are other opportunities for program improvement. Quality assurance/quality improvement activities serve two critical purposes: to improve individual care and to refine the transition model. These matters are discussed in the literature on measuring outcomes of health care transition efforts (3, 7, 69) but generally do not receive as much attention in the related literature that specifically addresses remedying transition-related barriers. These activities should cover all key aspects of the transition effort, including clinic operations and clinical effectiveness. Examples include wait times, timeliness of transfer, gaps in care such as missed appointments, preventative and medication care management, insurance continuity, cost effectiveness, and longitudinal outcomes and should involve feedback from all parties, especially that of persons with IDD and other key stakeholders. The standardized inclusion of quality assurance/quality improvement processes in pediatric to adult health care transition programs would likely result in multiple significant benefits for patients and the overall transition program.

Moreover and as noted above, relatively recent data indicate disparities based on race, ethnicity, socioeconomic status and diagnosis regarding the transition from pediatric to adult health care for persons with IDD (8, 24, 25, 51). Consequently, another vital part of a transition program's quality assessment/quality improvement process should be to assess its effectiveness in achieving equitable health care access and outcomes for all adolescents and young adults with IDD, as has been proposed recently (69–71).

Another set of potential opportunities for transition program improvement involves the consideration of the team that works with the patient and his/her caregivers. There are varying designs of successful health care transition programs including, for example, “general” transition clinics, organ system- or disorder-specific transition clinics, and different permutations of consultation transition clinics or transition teams. Staffing, therefore, will necessarily be contingent on the needs of the clinic population, the design of the transition program, and the local availability of personnel. This should include representation from primary care and major specialties pertinent to the patient, as well as community health or social workers who are able to address and provide resources for the medical issues and social factors affecting patients' well-being.

Among specialist care, it is imperative for patients to receive adequate mental health support, especially since mental health often declines during transition (34, 72, 73). The involvement of mental health professionals as central members of a transition program is an important consideration.

The dental needs of all persons with IDD must be met and ideally should be considered within the context of a transition program. As with medical transitional care, there are multiple barriers in obtaining dental transitional care. These include a shortage of general dentists who are willing and able to provide dental care for those with special health care needs and reimbursement-related barriers (74–76). Patients' dental care during transition should not be considered apart from their other medical needs.

Medical geneticists are not commonly included as part of the core health care transition team for those with IDD. This is sometimes because medical geneticists, if part of transition teams, are mainly used in consultative roles in genetic disorder-specific transition programs. In some instances, this may be due to unavailability of such clinicians. Yet these specialists, when available, can serve multiple useful functions in transition programs. A considerable percentage of those with IDD have an underlying genetic etiology for their condition (77, 78). Medical geneticists can assist in the diagnostic evaluation of the many persons with IDD who do not have an etiologic diagnosis. A genetic evaluation can sometimes reveal diagnoses that in their absence would deprive a patient of a useful treatment, such as in some persons mistakenly diagnosed with cerebral palsy (78). Moreover, persons with uncommon or rare cytogenomic or monogenic conditions with IDD can benefit from the inclusion of medical geneticists on the care team because medical geneticists have familiarity with the natural histories and management of these rare conditions. There are means to incorporate the input of medical geneticists into the primary care of the patient even if they are unable to be physically present (79). Genetic counselors can similarly have helpful roles in health care transition programs (80).

Another potential opportunity relates to the use of short videos. Patients, caregivers and clinical providers all reported on difficulties in the communication of clinical information about patients to clinical providers in the adult medical system. An approach that can augment existing methods and that, to our knowledge, is largely unused for this purpose is the use of short videos that contain pertinent medical and non-medical information about the patient. One possible scenario could be the production of two short, inexpensive videos. One of the videos would feature the patient and describe the patient's interests, likes and dislikes, hopes for the future and other general matters and that would be communicated by the patient, if possible, and/or his caregivers. We previously described the rationale and production of a similar video for adults with IDD who are about to live in out-of-home placements (81). A second short video could be made by a pediatric primary care clinician that would contain the summary of essential medical information needed for the adult care team that will be assuming care of the young adult with IDD. This film could include the patient and family as well, if desired.

Another, non-mutually exclusive method of ensuring understanding and communication of a patient's past and current medical condition is the utilization of a “warm handoff” between providers (32). This conversation can be done via telehealth, phone or even in person in a shared clinic visit with both providers, if circumstances allow. This is a method that we have employed, and our patients and caregivers have expressed their appreciation and reassurance following these interactions.

Finally, there are many unanswered questions regarding how best to accomplish effective pediatric to adult health care transitions for persons with IDD and their families. Some important issues include: What are the best health care transition models for persons with IDD and which models are best in consideration of different contexts (e.g., different health care systems, medical resource-rich vs. resource-poor areas, differing IDD clinical phenotypes)? What are the best approaches for integrating health care across community and government health care resources? How can the vocational, social and recreational needs of youth with IDD best be integrated into their pediatric to adult health care transition? What are the best ways to train the next generation of health care providers to become effective clinical practitioners in their areas of health care transition medicine? How can various communication technologies be used to enhance learning and empower persons with IDD to optimize their care? What are the ways that one can measure the different aspects of the health care transition process, including outcomes and quality assurance/quality improvement processes, and which measures are most useful? What are the best systems of region and country-specific government supports to assure effective, sustainable programs? These and many other relevant issues should be addressed through well-designed research studies, many of which have been nicely framed by others (69, 82). Because of the importance of these issues, health care transition programs should endeavor to have a research component to their operation. This, in turn, will likely fuel new and possibly unexpected advances in the care of this vulnerable population.

## Data Availability

The original contributions presented in the study are included in the article/Supplementary Material, further inquiries can be directed to the corresponding author.
