# Supplementary material for: Adolescent to adult health care transition for persons with intellectual and developmental disability: current barriers, next steps
Source: Front Pediatr. 2025 Aug 15;13:1486325. doi: 10.3389/fped.2025.1486325 (PMC12394008; doi:10.3389/fped.2025.1486325)
Supplement: Supplementary file 1 [file Datasheet1.pdf]

Supplementary Table 1: Barriers to Effective Pediatric to Adult Health Care Transition for Persons with IDD		
Health Care Transition Constraints[1]	Cause(s)[1]	Possible Ways to Remediate[1,2]
Inadequate availability of formal, structured pediatric to adult health care transition programs	• Low prioritization of transition programs<br>• Inadequate number of primary care and specialist clinicians with expertise or willingness to care for those with IDD<br>• Inadequate number of providers with expertise to address the unique needs of youth/young adults<br>• Inadequate remuneration for providers' services	• Integrate health care transition into routine primary, specialty and behavioral health care<br>• Improve communication of the need for pediatric to adult transition programs<br>• Increase programmatic investment<br>• Increase the number of clinical providers<br>• Improvement of all clinical curricula<br>• Reform of clinical service reimbursement system<br>• Region/country-specific legislation ensuring successful transfer of care<br>• Further research on models/outcomes of transition programs
Delayed entry in transition planning for patients/caregivers	• Inadequate policy/function of transition programs<br>• Variability of patients/caregivers in willingness to begin transfer<br>• Variability between providers regarding age of transfer	• Implement more and better programs<br>• Standardized and enforced upper age for transfer of care by medical providers
Emotionally difficult transition from pediatric care for patients/caregivers	• Absent transition programs	• Improve communication with family

	• Inadequate transition programs with disorganized communication among family and care team<br>• Lack of specialized social work providers	• Implement more and better programs<br>• Develop/utilize peer and caregiver supports
Inadequate number of primary care and/or specialist clinicians in the adult medical system with expertise in caring for those with IDD	• Inadequate teaching regarding IDD in medical and nursing schools, residencies and fellowships	• Improve all clinical curricula
Difficulty accessing primary care and/or specialist clinicians in the adult medical system with willingness to care for patients with IDD	• Ableism<br>• Inadequate remuneration for services	• Increase learning re: IDD and disability-related matters<br>• Reform of clinical service reimbursement system
Difficulties for patients/caregivers in making appointments	• Ableism<br>• Limited clinic resources	• Ensure accessibility scheduling processes (e.g., improve websites and phone systems)<br>• Make online chats or live help via phone available
Challenges for patients/caregivers regarding transportation to appointments	• Ableism<br>• Poverty/income inequality<br>• Geographic locale	• Improve access to transportation services<br>• Increased utilization of mobile clinical units, if available<br>• Increased utilization of telemedicine, when appropriate
Difficulties in the transfer of medical information from pediatric to the adult medical practices	• Inadequate electronic forms and portals<br>• Incompatible electronic communication systems<br>• Lack of education for clinical providers on available tools	• Develop improved electronic forms/portals<br>• Increase use of 'Care Everywhere' or similar tools<br>• Create written medical transfer summaries that are provided to patients/families

	• Insufficient time/reimbursement for discussion between pediatric clinicians and clinicians in the adult system	• Use short, patient-centered 'medical summary' videos<br>• Use short, patient-centered life interests/lived experiences videos<br>• Reform the clinical service reimbursement system<br>• Embed tools regarding health care transition in the EMR to prompt usage and track progress
Inadequate information about the transition process and related resources for patients/caregivers	• Inadequate transition programs	• Develop paper- and web-based resources for patients/families<br>• Create transition consult clinic to provide access to transition navigator<br>• Consultation with Legal Aid or embedded legal representation and/or social work in transition planning<br>• Utilization of peer supports
Inequitable access for patients related to the physical design of clinical spaces	• Ableism<br>• Limited institutional resources	• Follow ADA requirements (at a minimum)<br>• Subsidization for increasing accessible spaces
Insufficient information for clinicians in the adult medical system about the transfer process and care for persons with IDD	• Inadequate transition program<br>• Inadequate teaching in medical and nursing schools, residencies, fellowships<br>• Absent or inadequate professional practice policies	• Improve transition readiness materials for clinicians<br>• Improve clinical curricula<br>• New or improved professional practice policies

Variable self-advocacy skills of patients	• Inadequate individualized transition plan	• Improve transition program<br>• Utilization of peer supports, when possible<br>• Utilization of clinical psychologists or specialized social work providers
Inadequate duration of many appointments from patients'/caregivers'/clinical providers' perspectives	• Biased medical compensation practices (oriented to RVUs and productivity goals vs. medical education, preventive care, collaborative care, care for those with medical complexity)	• Improve receipt/review of patient/family information prior to clinic visits<br>• Use of scribes (actual person or AI)<br>• Extend clinic hours (evenings, weekends)<br>• Improve collaboration with medical system administration<br>• Reform of clinical service reimbursement system
Competing family and work obligations faced by caregivers during the transition process	• Excessive demands on caregivers<br>• Inadequate provider availability<br>• Scheduling conflicts between caregivers and needed services<br>• Child care needs	• Improved transition program with better navigation, coordination and services<br>• Provide childcare<br>• Adjust schedules of day programs to that of full-time workers
Negative impacts on caregivers' health related to the demands of managing their children's health needs	• Excessive demands on caregivers' time due to inadequate transition program<br>• Inadequate time for self care	• Improved transition program with better navigation, coordination and services<br>• Novel medical practice design to provide care to young adult and the adult caregiver(s) at the same visit
Challenges in handling issues relating to relinquishing or sharing	• Inadequate information for all parties	• Increase resources (legal, social work, psychol.)

medical decision making with patients	• Inadequate time for quality discussions<br>• Inadequate resources	• Reform of clinical service reimbursement system
Difficulties in communication between pediatric clinicians and clinicians in the adult system	• Biased medical compensation practices<br>• Inadequate time for quality discussions<br>• Incompatible electronic communication systems	• Reform of clinical service reimbursement system<br>• Increase allowance of time in clinical encounters<br>• Increase utilization of 'Care Everywhere' or similar tools
Difficulties in communication between clinicians in the adult medical system	• Biased medical compensation practices<br>• Inadequate time<br>• Incompatible electronic communication systems	• Reform of clinical service reimbursement system<br>• Increase allowance of time for this purpose<br>• Increase utilization of 'Care Everywhere' or similar tools
Inadequate or insufficient supplies or equipment for patients after transfer to the adult medical system	• Ableism<br>• Limited clinic resources<br>• Lack of clear transfer plan	• Follow ADA requirements (at a minimum)<br>• Implementation of improved transition plan
Inadequate assistance for patients/caregivers in navigating the health care transition process and in navigating the adult medical system	• Inadequate 'navigation' support of the transition team	• Implement a navigator or navigation team<br>• Utilization of peer/caregiver supports
Financial challenges for patients/caregivers related to clinical care and related services	• Expensive medical care	• Health insurance reform, incl. more emphasis on preventive and continued care<br>• Transition call number to triage need for seeing provider vs. resolving clinical matter at home
Inadequate reimbursement for clinical providers for their medical services	• Inadequate insurance coverage for preventative care and education	• Expand coverage of transition services

		• Educate providers regarding coding/billing for transition services<br>• Implement funding and payment models that are flexible regarding age cut-offs and shared among pediatric and adult health services<br>• Develop payment approaches to encourage collaboration between pediatric and adult care clinicians in the health care transition process
Inadequate financial and administrative support by institutions for their transition programs	• Low prioritization of transition programs<br>• Challenges in defining metrics for measurement of success in transition	• Improve allocation of resources by administrators<br>• Improve communication of value of transition programs<br>• Research on models and outcomes of transition programs.
Inadequate integration of education system, social and recreation needs, employment considerations, community systems/supports and legal considerations with the medical system	• Siloed operation of different systems<br>• Lack of coordination between public service agencies<br>• Lack of specialized social work providers	• Co-participation of pediatric and adult medical leadership, social work and others in transition program steering committees<br>• Inclusion of transition process in the education system, including IEP goals<br>• Integrate health care transition supports in legal discussion (eg, guardianship, power of attorney)<br>• Collaboration across sectors, such as health, education and social domains, that includes

		integration with local government services<br>• Train increased numbers of specialized social work providers<br>• Utilization of 'capacity building' model for transition program<br>• Increase employment options and the number of day programs for those unable to work
Inequitable access to quality pediatric to adult health care transition services	• Poverty/income inequality<br>• Discrimination based on race/ethnicity<br>• Ableism/discrimination based on diagnosis<br>• Maldistribution of health care resources and access related to geographic locale of the patient/family	• Equitable access of health care regardless of income/race/ethnicity/gender<br>• Include equity as an outcome measure of transition programs<br>• Include disability-related learning in clinical curricula, as well as in continuing education<br>• Increase use of telemedicine, mobile units and incentives to work in underserved areas<br>• Develop accessible health care facilities that includes input from disabled persons<br>• Provide ongoing disability training to health care workers

1) It should be noted that not all of the listed barriers, their causes or the proposed interventions are universally present or applicable.
2) Quality assurance/quality improvement processes are necessary aspects of most of the listed remediation efforts
